# Nivolumab-induced hypophysitis followed by acute-onset type 1 diabetes with renal cell carcinoma: a case report

**DOI:** 10.1186/s13256-020-02656-7

**Published:** 2021-04-23

**Authors:** Fumi Kikuchi, Takanobu Saheki, Hitomi Imachi, Toshihiro Kobayashi, Kensaku Fukunaga, Tomohiro Ibata, Seisuke Sato, Natsuki Ban, Jingya Lyu, Salimah Japar, Koji Murao

**Affiliations:** 1 Department of Endocrinology and Metabolism, Faculty of Medicine, Kagawa University, 1750-1, Miki-cho, Kita-gun, Takamatsu, Kagawa 761-0793 Japan; 2 Present Address: Department of Internal Medicine, Sanuki Municipal Hospital, 387-1, Sangawa-cho, Sanuki, Kagawa 769-2393 Japan

**Keywords:** Immune checkpoint inhibitor, Hypophysitis, Acute type 1 diabetes mellitus, Immune-related adverse events, Nivolumab

## Abstract

**Background:**

Immune checkpoint inhibitors have recently become widely used for the management of advanced cancer patients. During the development of immune checkpoint inhibitors (ICPIs), it was quickly recognized that they are associated with autoimmune or autoinflammatory side effects. These toxicities are known as immune-related adverse events (irAEs): common endocrine irAEs include hypophysitis and thyroid dysfunction, and uncommon irAEs include type 1 diabetes mellitus (T1DM).

**Case presentation:**

A 62-year-old Japanese man with metastatic renal cell carcinoma was treated with sunitinib followed by the 10th cycle of treatment with the ICPI nivolumab. He had already had thyroiditis and hypophysitis due to these anti-cancer drugs. On admission, he showed an extremely elevated plasma glucose level (601 mg/dl) and a low C-peptide level, and was diagnosed with acute T1DM. The patient was treated with intravenous fluid infusion and continuous insulin infusion. On the second day, he was switched to multiple daily injections of insulin therapy. Since these treatments, his blood glucose levels have been stable and he has been treated with an additional 10 ICPI treatments for renal cell carcinoma for over a year.

**Conclusions:**

Treatment with ICPIs is expected to increase in the future. There may be cases in which their use for cancer treatment is inevitable despite the side effects. As long as treatment with ICPI continues, multiple side effects can be expected in some cases. It is important to carefully observe the side effects that occur during ICPI treatment and to provide appropriate treatment for each side effect.

## Background

The efficacy of immune checkpoint inhibitors (ICPIs) has led to their widespread use for the management of advanced cancer patients. Nivolumab is a typical ICPI that is an anti-programmed cell death protein 1 (PD-1) antibody designed to promote an immunologic reaction against cancer cells including melanoma, non-small cell lung cancer, and renal cell carcinoma (RCC) by blocking the activation of the PD-1-mediated pathway [1]. The PD-1 protein is a cell-surface molecule on T cells that prevents the activation of antigen-specific T cells, including those directed against tumors. During the development of ICPIs, it was quickly recognized that they are associated with autoimmune or autoinflammatory side effects [2]. These toxicities are known as immune-related adverse events (irAEs): common endocrine irAEs include hypophysitis and thyroid dysfunction, and uncommon irAEs include type 1 diabetes mellitus (T1DM). The factors that predict irAEs remain unclear. ICPI-related hypophysitis is frequently (up to 17% of cases) associated with ipilimumab, an anti-cytotoxic T lymphocyte-associated antigen-4 (CTLA-4) antibody, but hypophysitis is an extremely rare event (< 1%) in patients treated with other ICPIs, such as nivolumab [3]. In contrast, nivolumab-related T1DM reportedly manifests as fulminant type 1 diabetes mellitus (FT1DM), which is an emergency condition, as patients develop ketosis or ketoacidosis within approximately 1 week [4].

Here, we describe the case of a patient with metastatic renal cell carcinoma (mRCC) who developed T1DM followed by hypophysitis during nivolumab therapy.

## Case presentation

**Fig. 1:**
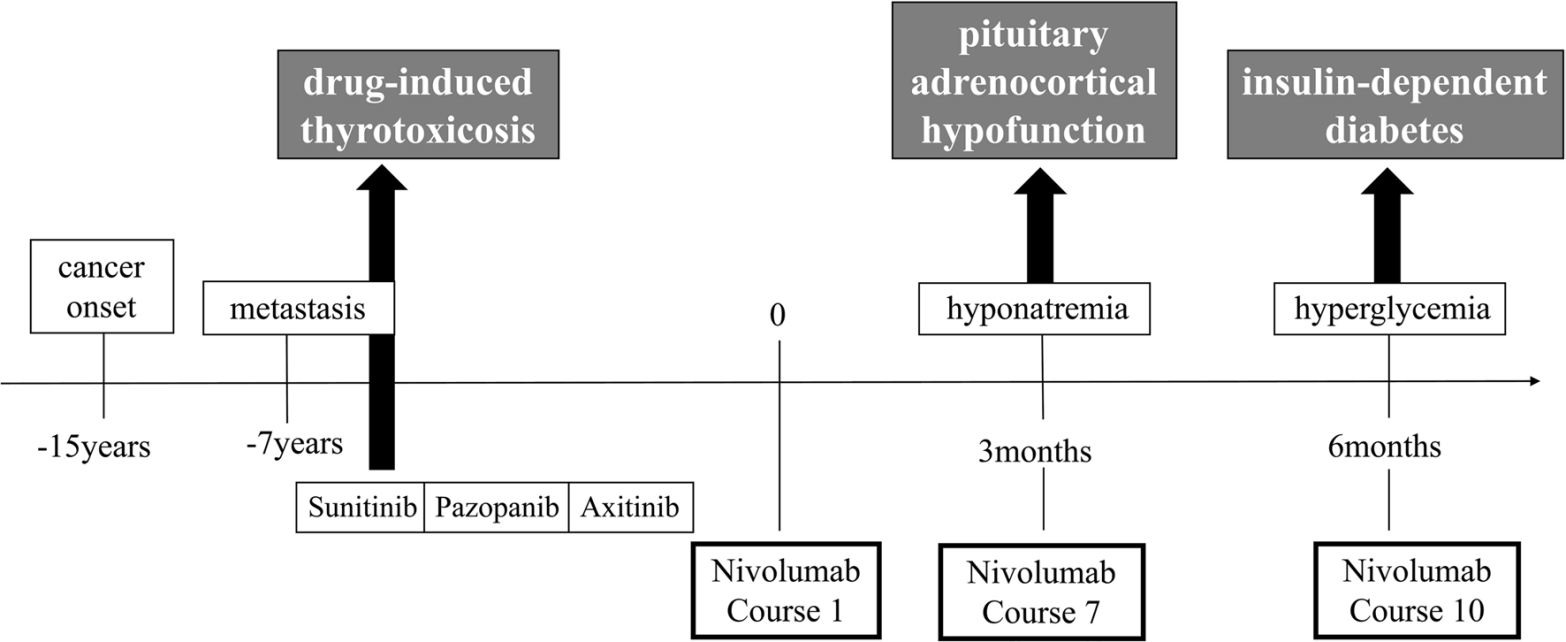
The clinical course of multiple lines of chemotherapy and nivolumab

A 62-year-old Japanese man was referred to our department by the urology department because of hyponatremia. In 2003, he was diagnosed with right renal cancer, and he underwent right nephrectomy. Seven years later, a cancerous lesion was found in the right clavicle. Subsequent bone biopsy revealed that it was caused by metastasis of the renal cancer. He then underwent multiple rounds of chemotherapy (Fig. 1), and developed thyrotoxicosis after starting sunitinib treatment. Sunitinib treatment was discontinued, and the patient has been treated with the ICPI nivolumab (3 mg/kg) every 2 weeks since 2018.

After administration of the seventh cycle of nivolumab treatment, the patient complained of fatigue and dizziness, and was admitted to the urology department. On physical examination, the patient’s blood pressure was 93/59 mmHg, his pulse was 73 beats per minute, and his body temperature was 36.5 °C. His weight and height were 56.3 kg and 165 cm, respectively. Results of the laboratory tests are shown in Table 1. The patient had hyponatremia (120 mmol/l), with low levels of cortisol (0.4 μg/dl) and adrenocorticotropic hormone (ACTH) (6.64 pg/ml), suggesting adrenal insufficiency. The serum levels of luteinizing hormone (LH) (11.4 mIU/ml) and prolactin (PRL) (38.5 ng/ml) were high, and those of insulin-like growth factor (66 ng/ml) were low. His symptoms and laboratory data suggested that the clinically suspected adrenal insufficiency was possibly due to pituitary adrenocortical hypofunction induced by nivolumab immunotherapy, despite the absence of changes in the size and density of the pituitary gland revealed on brain magnetic resonance imaging (MRI). The patient was started on steroid replacement therapy with 40 mg/day prednisolone. After starting steroid therapy, his physical condition improved rapidly. The dose of steroid was tapered and was finally maintained at 20 mg/day of hydrocortisone. The patient became hyperglycemic because of the steroid treatment, which was managed with insulin. Because the ability to secrete insulin was unimpaired (C-peptide immunoreactivity [CPR] 2.37 ng/ml), he was started on liraglutide therapy, and nivolumab treatment was continued. After his symptoms subsided, the corticotropin-releasing hormone (CRH) load test was performed, which revealed that the serum ACTH level and cortisol response to CRH were very low (Table 2).

**Table 1:** Laboratory results for the patient

Variable		Reference range
Complete blood count		
White blood cell count (/μl)	5160	47.0–87.0
Red blood cells (×10^4^/μl)	383	370–490
Hemoglobin (g/dl)	9.8	11.0–15.0
Hematocrit (%)	29.4	35.0–45.0
Platelet count (×10^4^/μl)	30.4	15.0–35.0
Biochemistry		
Total protein (g/dl)	6.1	6.5–8.2
Albumin (g/dl)	3.1	3.5–5.5
Blood urea nitrogen (mg/dl)	11.4	7.0–20.0
Creatinine (mg/dl)	1.46	0.5–1.0
AST (U/l)	38	10–35
ALT (U/l)	13	5–40
GGTP (U/l)	17	0–30
Na (mmol/l)	120	135–146
K (mmol/l)	4.6	3.5–4.6
Cl (mmol/l)	88	96–110
Plasma osmolality (mOsm/kg)	292	275–295
Diabetes-related examination		
Glucose (mg/dl)	112	70–109
HbA1c (on admission) (%)	7.0	4.6–6.2
Serum CPR (ng/ml)	2.37	0.43–2.35
Endocrinological examination		
ACTH (pg/ml)	6.64	7.2–63.3
Cortisol (μg/dl)	0.4	2.3–19.4
GH (ng/ml)	1.61	0.28–1.64
IGF-1 (ng/ml)	66	76–228
PRL (ng/ml)	38.5	4.3–13.7
LH (mIU/ml)	11.4	0.57–12.07
FSH (mIU/ml)	7.6	0.95–11.95
Testosterone (ng/ml)	477.5	142.4–923.1
AVP (pg/ml)	0.8	0.3–4.2
TSH (μIU/ml)	2.14	0.35–4.94
FT3 (pg/ml)	3.71	1.71–3.71
FT4 (ng/dl)	1.03	0.70–1.48
Urinalysis		
U-Osmolality (mOsm/kg)	178	767–1628
U-Na (mmol/l)	28	
U-K (mmol/l)	17	
U-Cl (mmol/l)	29	
U-Creatinine (mg/dl)	29	

*AST* aspartate aminotransferase, *ALT* alanine transaminase, *GGTP* gamma-glutamyltransferase, *HbA1c* hemoglobin A1c, *CPR* C-peptide immunoreactivity, *ACTH* adrenocorticotropic hormone, *GH* growth hormone, *IGF1* insulin-like growth factor, *PRL* prolactin, *LH* luteinizing hormone, *FSH* follicle-stimulating hormone, *AVP* arginine vasopressin, *TSH* thyroid-stimulating hormone, *FT3* free triiodothyronine, *FT4* free thyroxin

**Table 2:** Laboratory results of corticotropin-releasing hormone tests

Min	0	30	60	90
ACTH (pg/ml)	1.0	1.0	1.0	1.0
Cortisol (µg/dl)	0.8	0.7	0.6	0.5

*ACTH* adrenocorticotropic hormone

**Fig. 2:**
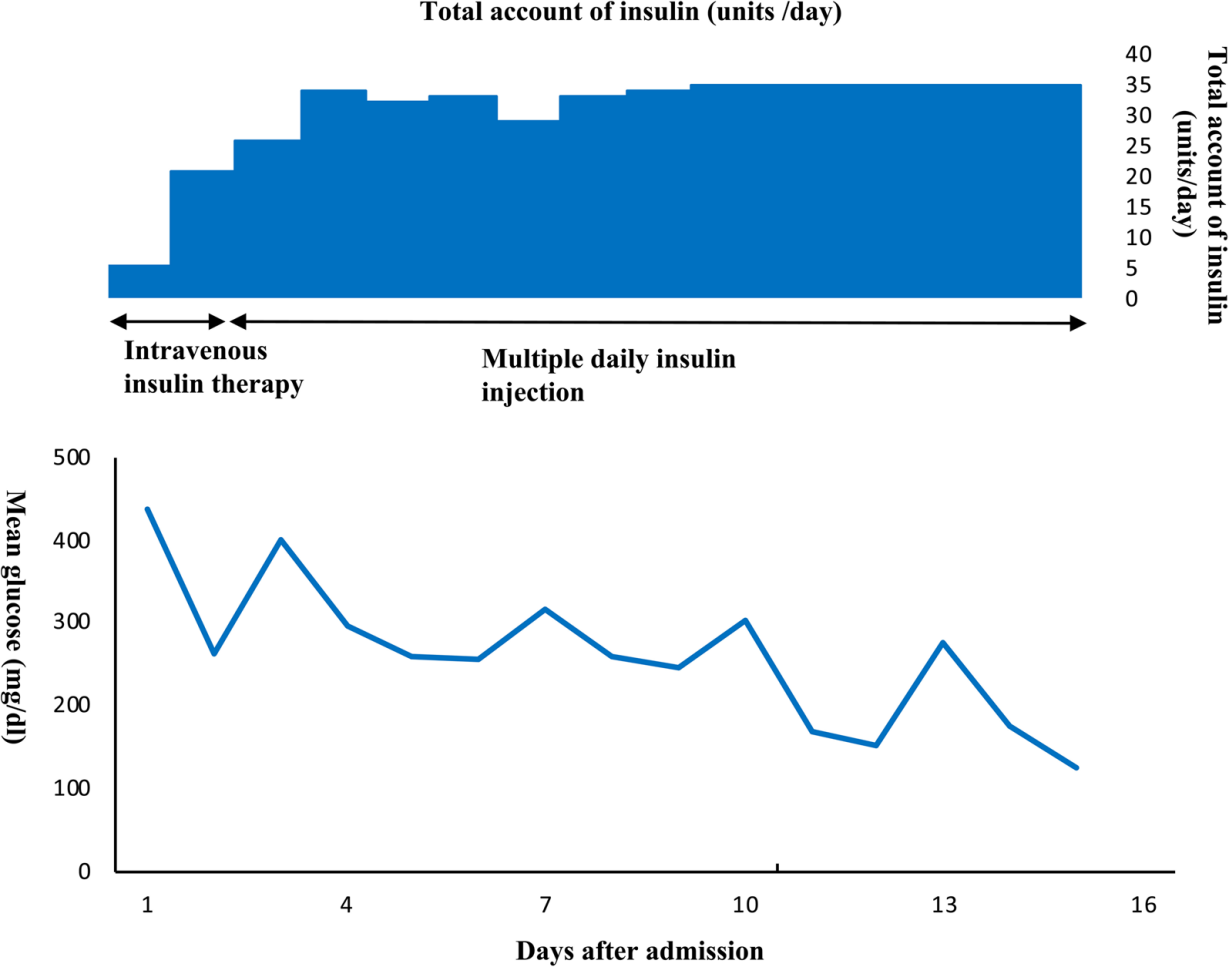
The clinical course of insulin therapy. The upper panel shows the total insulin dose. The lower panel shows the mean glucose levels during the clinical course

Following the 10th cycle of nivolumab treatment, the patient was referred to our department again because of high blood sugar levels. He was admitted to the hospital for suspected FT1DM associated with nivolumab treatment and was started on drip infusion therapy. On physical examination, the patient’s blood pressure was 148/72 mmHg, his pulse was 75 beats per minute, and his body temperature was 36.3 °C. His mouth was dry and his skin turgor was poor, but he did not complain of any other symptoms. Although his plasma blood glucose levels were high (601 mg/dl), his hemoglobin A1c (HbA1c) levels were low (8.1%), suggesting rapid progression of hyperglycemia. Abdominal computed tomography showed no pancreatic abnormality. Both his serum C-peptide levels and his urinary C-peptide excretion levels were low. A glucagon stimulation test revealed insulin depletion, but he showed serum anti-glutamic acid decarboxylase (GAD) antibody negativity (details are outlined in Table 3). Laboratory investigations revealed hyperglycemia, but no acidosis or ketonuria. On the basis of these findings, we excluded FT1DM, and diagnosed the patient with insulin-dependent diabetes. The patient was treated with intravenous fluid infusion and continuous insulin infusion. His potassium and other electrolyte levels were within the normal range, and serum potassium fluctuations were within the normal range even after the introduction of insulin therapy. On the second day, he was switched to multiple daily injections of insulin therapy (Fig. 2). Since these treatments, his blood glucose levels have been stable and he has been treated with an additional 10 ICPI treatments for RCC for over a year.

**Table 3:** Laboratory results for the patient

Variable		Reference range
Complete blood count (on admission)		
White blood cell count (/μl)	6500	47.0–87.0
Red blood cells (×10^4^/μl)	425	370–490
Hemoglobin (g/dl)	11.6	11.0–15.0
Hematocrit (%)	34.5	35.0–45.0
Platelet count (×10^4^/μl)	36.5	15.0–35.0
Biochemistry (on admission)		
Total protein (g/dl)	6.1	6.5–8.2
Albumin (g/dl)	2.8	3.5–5.5
Blood urea nitrogen (mg/dl)	31.7	7.0–20.0
Creatinine (mg/dl)	1.64	0.5–1.0
Amylase (U/l)	168	30–140
Lipase (U/l)	28	13–55
Elastase-1 (ng/dl)	110	0–300
AST (U/l)	25	10–35
ALT (U/l)	20	5–40
GGTP (U/l)	76	0–30
Na (mmol/l)	131	135–146
K (mmol/l)	4.5	3.5–4.6
Cl (mmol/l)	97	96–110
Arterial blood gas analysis (room air)		
pH	7.44	7.35–7.45
pCO_2_ (mmHg)	37.2	32–45
pO_2_ (mmHg)	113	83–108
Bicarbonate (mmol/l)	24.7	21.2–27.0
Anion gap (mmol/l)	8.2	10.0–14.0
Diabetes-related examination		
Glucose (mg/dl)	601	70–109
HbA1c (3 months before admission) (%)	7.0	4.6–6.2
HbA1c (on admission) (%)	8.1	4.6–6.2
Serum CPR (ng/ml)	0.2	0.43–2.35
Urinary CPR (μg/day)	5.3	17–181
CPR (6 min) during glucagon stimulation test (10th day) (ng/ml)	0.29	> 0.5
Urinary ketone	Negative	
Anti-GAD antibody	Negative	

*AST* aspartate aminotransferase, *ALT* alanine transaminase, *GGTP* gamma-glutamyltransferase, *pCO*_*2*_ partial pressure of carbon dioxide,  *pO*_*2*_ partial pressure of oxygen, *HbA1c* hemoglobin A1c, *CPR* C-peptide immunoreactivity, *GAD* glutamic acid decarboxylase

## Discussion

We presented the case of a patient with mRCC who developed thyroiditis on sunitinib treatment, which was changed to nivolumab therapy that caused hypophysitis and subsequently T1DM.

Sunitinib, a tyrosine kinase inhibitor (TKI), belongs to a class of drugs mainly used as targeted therapy in cancer treatment [5]. TKIs typically compete with adenosine triphosphate (ATP) for the binding site of particular oncogenic tyrosine kinases. By blocking the signaling pathways involved in the phosphorylation of many key proteins in signal transduction cascades, TKIs can depress tumor cell survival and proliferation. TKI-induced hypothyroidism is a highly complicated issue not only because of the unclear toxicological mechanisms, but also because of the different incidence of individual TKI drugs. While sunitinib is suspected of causing thyroid dysfunction more often than other TKIs, sorafenib is believed to be less risky [6]. In our case, nivolumab was used in anticipation of further effects.

An estimated 20–30% of all patients with RCC have metastatic disease at the time of diagnosis. A recent study demonstrated the safety and acceptable outcomes of anti-PD-1 therapy with nivolumab in patients with advanced RCC. Although nivolumab caused unique AEs, the AEs were treated promptly, and all except one was reversible. A recent study suggested that nivolumab is a reasonable therapeutic option for patients with mRCC after multiple lines of treatment [7]. However, future prospective studies with larger sample sizes are required to better understand the appropriate indications for nivolumab treatment in patients with RCC.

ICPI-related hypophysitis is frequently (up to 17% of cases) associated with the use of ipilimumab, an anti-CTLA-4 antibody, and patients with ipilimumab-induced hypophysitis typically experience headache, multiple anterior pituitary hormone defects, and reversible enlargement of the pituitary gland [8]. In contrast, hypophysitis is an extremely rare event (<1%) in patients treated with other ICPIs, such as nivolumab [9]. The thyroid gland is the most commonly affected endocrine organ in patients under nivolumab treatment [1]. For example, only 18 hypophysitis cases (0.9%) were identified in 10 studies that included approximately 2000 patients receiving nivolumab, pembrolizumab, or anti-programmed death-ligand 1 (PD-L1) [2]. Furthermore, nivolumab-associated all-grade pituitary dysfunction occurred in only 0.5–0.9% of reported cases [8], and there are only three related case reports [10]. Radiographic pituitary enlargement was detected in only one of four cases, suggesting that morphologic changes in the pituitary may be less severe in patients with nivolumab-induced hypophysitis than that in patients with ipilimumab-induced hypophysitis. A previous report described dysfunction in the secretion of thyroid-stimulating hormone (TSH), ACTH, LH/follicle-stimulating hormone (FSH), and PRL in 92.0%, 74.0%, 85.7%, and 67.7% of ipilimumab-induced hypophysitis cases, respectively [8], whereas dysfunction in TSH secretion was only observed in two of four nivolumab cases, and no cases exhibited dysfunction in LH/FSH secretion. This information, along with the radiographic characteristics, suggests that the inflammatory process in the pituitary gland is much milder in nivolumab-induced hypophysitis than in ipilimumab-induced hypophysitis. In our case, there were no abnormal pituitary findings on MRI. As a side effect of nivolumab, hypophysitis was unusual but could be treated with symptomatically. In our case, even though there were side effects, nivolumab treatment was indispensable because it was related to life prognosis of mRCC.

Anti-PD-1 antibodies activate an anti-tumor immunologic response by abrogating PD-1-related T cell inhibition. They reportedly improve the prognosis of patients with several advanced malignancies [1]. Although nivolumab, an anti–PD-1 antibody, has improved the prognosis of and has become a popular treatment option for several advanced malignancies, various irAEs [11], including diabetes, have been reported. Although the precise mechanisms of nivolumab-induced T1DM are not fully understood, activated CD8+ T cells have been speculated to evoke autoimmunity against pancreatic beta cells and to destroy them, resulting in insulin exhaustion [12]. Several reports have noted that cases of autoimmune diabetes, known as T1DM, have emerged in association with the use of the anti-PD-1 antibody therapies [5, 13]. Autoimmune diabetes is characterized by the development of an adaptive immune response against specific beta cell antigens. Longitudinal studies in patients have shown that certain autoantibodies, such as anti-indole 3 acetic acid (IAA), anti-islet cell antigen (ICA) 512, and anti-GAD65, define preclinical disease, as they are present in the serum for years before symptom onset. Likewise, half of the previously reported patients who developed insulin-dependent diabetes after anti-PD-1 therapy showed no detectable islet autoantibodies. The pathogenesis in these patients thus seems to differ at least partly from that of conventional autoimmune T1DM, which involves islet autoantibodies. Ansari *et al.* found no correlation between IAA levels and the development of diabetes with blockade of the PD-1/PD-L1 pathway in mice, and some mice developed diabetes despite the apparent absence of autoantibodies [14]. In the present case, the patient tested negative for islet autoantibodies.

FT1DM has mainly been reported in East Asia, and it accounts for approximately 20% of T1DM cases with a classical acute-onset pattern in Japan [15]. According to the safety database, 20,600 patients in Japan were treated with nivolumab from July 4, 2014, to August 15, 2017, and 67 patients (0.33%) developed “T1DM” or “FT1DM.” Among these, 40 patients (0.19%) were reported as having T1DM (10 patients had melanoma, 24 patients had non-small cell lung cancer, 3 patients had RCC, and the diagnosis for 3 patients was unknown), and 27 patients (0.13%) were reported as having FT1DM (14 patients had melanoma, 12 patients had non-small cell lung cancer, and the diagnosis for 1 patient was unknown) [16]. Therefore, the incidence rate of T1DM related to nivolumab is 33 times as high as that of typical FT1DM. In addition, there was no particular relationship between primary tumors and anti-PD-1 antibody-related T1DM.

Acute-onset T1DM was diagnosed based on the criteria set by the Committee of the Japan Diabetes Society (JDS) [17]. Briefly, among patients who developed diabetic ketosis or ketoacidosis within 3 months after the onset of hyperglycemic symptoms and patients who needed insulin treatment continuously after their diabetes diagnosis, patients with islet autoantibodies were diagnosed as having “acute-onset T1DM (A1DM) (autoimmune),” and those whose endogenous insulin secretion was depleted (fasting serum C-peptide immunoreactivity < 0.6 ng/ml) without verifiable islet autoantibodies were diagnosed as having “acute-onset type 1 diabetes.” The diagnosis of FT1DM was based on the following criteria from the Report of the JDS Committee on type 1 diabetes mellitus research: (1) ketosis or ketoacidosis within 1 week after the onset of hyperglycemic symptoms, (2) urinary C-peptide level below 10 μg/day or fasting serum C-peptide level below 0.3 ng/ml and serum C-peptide level below 0.5 ng/ml after glucagon injection or meal load soon after the onset of disease, and (3) plasma glucose (PG) level above 288 mg/dl and HbA1c below 8.7% at the first visit [18]. Although this case was diagnosed as A1DM on the basis of diagnostic criteria, it is very similar to FT1DM on the basis of the clinical course. Among the 35 published cases of nivolumab-induced T1DM, 17 patients were reported to be positive and 17 negative for islet autoantibodies, and the islet autoantibody levels were not measured in one patient. As in the present case, rapidly progressive T1DM was observed in eight patients without any detectable autoimmune islet antibodies; however, those patients were human leukocyte antigen (HLA)-type-positive, which resulted in sensitivity to acute-onset T1DM. Nivolumab may likely induce rapidly progressive T1DM through an autoimmune mechanism independent of conventional pancreatic islet cell-related autoantibodies [19].

The pathogenesis of FT1DM is influenced by genetic and environmental factors. Among genetic factors, HLA class II genes are strongly associated with susceptibility to progression to FT1DM. The HLA DR4-DQ4 haplotype is common in Japanese individuals but is rare in the Caucasian populations and may contribute to the different incidence rates of FT1DM between the these two populations. Imagawa *et al.* reported that the frequency of HLA-DR4, but not HLA-DR9, was significantly higher in FT1DM, whereas the frequencies of HLA-DR1, HLA-DR2, HLA-DR5, and HLA-DR8 were significantly lower. In contrast, DR9 but not DR4 was more frequent, and DR2 was extremely rare in T1DM [20]. Additionally, a recent report indicated that the frequencies of the DRB1*04:05-DQB1*04:01 and DRB1*09:01-DQB1*03:03 haplotypes were significantly higher and those of the DRB1*01:01-DQB1:05:01, DRB1:15:02-DQB1*06:01 and DRB1*08:03-DQB1*06:01 haplotypes were significantly lower in patients with FT1DM than in control subjects [21]. In our study, informed consent could not be obtained, and an HLA search could not be performed, but if an HLA search had been performed, it would have been a predictor of the onset of T1DM due to nivolumab. We believe that an HLA search should be performed in the future in patients who use nivolumab.

There are a few reports of patients who developed both hypophysitis and T1DM after being treated with an ICPI [22]. Several studies showed that panhypopituitarism induced a remarkable reduction in insulin doses in patients with T1DM [22, 23]. A complete amelioration of diabetes induced by adrenal insufficiency was reported in patients with type 2 diabetes [24], but it has never been reported in a patient with T1DM with decreased insulin secretory capacity to an insulin-dependent level as observed in our patient.

## Conclusions

ICPI treatment is expected to increase in the future. There may be cases in which their use for cancer treatment is inevitable despite the side effects. As long as treatment with ICPI continues, multiple side effects can be expected in some cases. It is important to carefully observe the side effects that occur during ICPI treatment and to provide appropriate treatment for each side effect.

## Data Availability

Not applicable.

## Abbreviations

ICPIs: Immune checkpoint inhibitors
irAEs: Immune-related adverse events
T1DM: Type 1 diabetes mellitus
PD-1: Anti-programmed cell death protein 1
CTLA-4: Anti-cytotoxic T lymphocyte-associated antigen-4
mRCC: Metastatic renal cell carcinoma
CRH: Corticotropin-releasing hormone
TKI: Tyrosine kinase inhibitor
A1DM: Acute-onset T1DM
